# Impact of phage treatment on fire blight disease outcome and floral microbiome composition

**DOI:** 10.1128/aem.01598-25

**Published:** 2025-10-15

**Authors:** D. Holtappels, K. U. Wu, B. Koskella, E. Roh

**Affiliations:** 1 Department of Integrative Biology, University of California, Berkeley 1438, Berkeley, California, USA; 2 Chan-Zuckerberg San Francisco Biohub 578083, San Francisco, California, USA; 3 Plant Disease Control Division, National Institute of Agricultural Sciences, Rural Development Administration, Wanju, Republic of Korea; The University of Tennessee Knoxville, Knoxville, Tennessee, USA

**Keywords:** biological control, microbiome, bacteriophage, fire blight

## Abstract

With the increasing importance of alternative pesticides for the control of bacterial pathogens in agricultural and clinical settings, the use of bacteriophage viruses (phages) to reduce bacterial growth and prevent disease is gaining in popularity. Phages have been shown to be highly effective in killing bacterial cells both *in vitro* and across plant and animal host systems, although many questions remain about the predictability of their success across more realistic ecological conditions and in light of natural strain variation of pathogens. Furthermore, as phage application becomes more common, it is imperative that we better understand the consequences of these treatments on the microbial communities associated with hosts (i.e., their microbiomes). Here, we leverage a recently developed phage cocktail targeting the causal agent of fire blight disease, *Erwinia amylovora*, to both test the efficacy of these phages in pear flowers inoculated with the pathogen and to ask whether such application has adverse effects on the resident microbiome of flowers. We found that phages are capable of greatly reducing both pathogen numbers and disease symptoms, but their application does not significantly alter the floral microbiome, emphasizing their high specificity to their target host. These data support the safe and effective use of phages in this disease system.

**IMPORTANCE:**

There is a critical need to develop new strategies to control bacterial diseases in crops, particularly to address the emerging problem of fire blight in pomme fruit. Bacteriophages, as viruses that infect and kill bacteria, are an appealing strategy. However, there is little information on how these viruses are impacting natural microbial communities, their potential off-target effects, and their environmental safety. This study addresses these questions, looking at the impact of an effective phage cocktail targeting *Erwinia amylovora*, the causal agent of fire blight in pomme fruit. In our research, we demonstrated that our phage cocktail does not affect the natural floral microbiome of Bradford pear blossoms. We further found that in the presence of *E. amylovora*, our phage cocktail significantly increases the richness and diversity of the microbial community. Our data, together with other studies performed in parallel, add to the small but growing evidence that phage application is unlikely to have an impact beyond the target bacterial pathogens they are used to treat.

## INTRODUCTION

*Erwinia amylovora* is a notorious plant pathogen and causal agent of fire blight in commercial pear and apple orchards. In recent years, outbreaks of fire blight have been reported all around the world, with devastating outcomes to growers (1). In South Korea, there is a relatively recent outbreak of fire blight (first reported in 2020) in South Korea with a total of 744 orchards demonstrating signs of the disease (2). The potential origin of the infection in previous pest-free regions of the country was determined to be the transport of infected plant material, after which the disease spread to neighboring orchards by natural means such as wind, rain, and insects, including pollinators. The latter are considered important vectors for *E. amylovora* as flowers are a critical entry point from where the infection establishes (1). As soon as the bacteria arrive and begin to grow on the flower stigma, they can enter the plant tissues via the hypanthium, from where they move systematically through the parenchyma to form biofilm, breaking the plant’s epidermis, which allows for bacterial “ooze” to escape the plant tissues, forming a secondary infection site. This ooze, in turn, can be effectively dispersed by insects that act as vectors to spread bacteria to neighboring plants (3).

Whether or not *E. amylovora* is successful in causing fire blight disease symptoms will depend on the genetic background of the pathogen, the plant, and the abiotic and biotic environment in which it occurs (4). Abiotic drivers, such as humidity and temperature, dictate the development of fire blight and the severity of the overall outbreak, which allows for the development of forecasting models (5). However, the biotic environment, such as the resident microbial community of flowers at the time of pathogen arrival, plays a critically important role as well. In healthy flowers, the microbiome primarily consists of bacteria belonging to the *Enterobacteriaceae* and *Pseudomonadaceae* and seems to be quite similar among different apple cultivars (6, 7). When *E. amylovora* is artificially inoculated into flowers, this community shifts as *Enterobacteriaceae* become more dominant in the microbial community (8). In addition to being naturally important in shaping the outcome of disease, these biotic interactions can be usefully leveraged as strategies to mitigate the outbreak of fire blight in commercial settings. In particular, there has been good progress in identifying and deploying specific microbes that directly compete with *E. amylovora* either through niche competition or the production of secondary/specialized metabolites. To date, several such bacterial isolates have been identified and commercialized, among which *Pantoea* sp. CT-1093, *Pantoea agglomerans* E325 (Bloomtime), *Pantoea vagans* C-1 (BlightBan C9-1), *Pseudomonas fluorescens* A506 (BlightBan A506), and *Bacillus subtilis* QST713 (Serenade). This approach has also been extended to disease-protective fungi such as *Aureobasidium pullulans* CF10 and CF40 and yeasts such as *Cystofilofasidium infirmominiatum* YY6 and *Cryptococcus neoformans* C9 and C16, all of which mitigate the severity at which fire blight emerges (1).

Thinking beyond microbial competition, the biotic environment also includes viruses, and many of these (phages) are capable of directly killing the bacterial pathogen (9) or altering the resident microbial community in a way that changes the competitive environment for the pathogen. Lytic bacteriophages infect and lyse bacterial cells in a strain-specific way and have been successfully applied for disease control across many plant disease systems, including fire blight (10–21). Worldwide, there are quite elaborate endeavors to isolate, characterize, and test different phages for their ability to control fire blight, as reviewed by Gayder and colleagues (22). These endeavors have resulted in several phage cocktails that are currently commercially available, albeit under strict regulations (22), but there are many questions around the environmental safety of these applications. Importantly, as phage host range remains a highly active area of research (23), it is often unclear whether phage application might have unintended impacts on the resident microbiome within a host. In the case where a phage targets not only the pathogen but also related, non-pathogenic strains/species of bacteria, their application could, in fact, remove/reduce populations of important competitor species from the environment, paving the way for growth and disease of the pathogen. It is thus critical to test not only the impacts of phages on their target pathogen but also to better understand the impacts on the broader host microbiome. A pioneering study focusing on the effects of a commercial phage product (AgriPhage) on the floral microbiome of apple blossoms in the presence of *E. amylovora* specifically demonstrated no adverse effects of the phage on the overall richness and composition of the microbial community (24).

In this study, we evaluate the potential of the FireFighter phage cocktail in mitigating the disease severity of *E. amylovora* in Callery pear flowers collected from Berkeley (CA). Callery pear is an ornamental pear tree planted across many urban areas and presents an excellent system for studying natural fire blight dynamics, as the trees are not treated against disease despite having active disease symptoms. The phage cocktail we used is composed of four different bacteriophages (ΦFifi011, ΦFifi44, ΦFifi287, and ΦFifi318, with GenBank accession numbers PQ051109, PQ051110, PQ051113, and PQ051114, respectively) belonging to different taxonomic groups. Fifi011 and Fifi287 both belong to the *Schitoviridae* family and the Waedenswilvirus genus and Yonginvirus genus, respectively. Based on homology, Fifi44 is part of the *Chaseviridae* family and *Cleopatravirinae* subfamily, and Fifi318 shows homology to the FelixO1-like cluster and is part of the Kolesnikviruses of the *Ounavirinae* family (25). The cocktail is efficient in reducing fire blight symptoms in saplings infected by Korean strains of *E. amylovora* (26). Furthermore, the cocktail is specifically designed to reduce the risks of cross-resistance between the different phages (26, 27). We determined the overall efficacy of this phage cocktail to control *E. amylovora* infections in pear blossoms across different pathogen densities, measuring reduction in disease symptoms and pathogen density. Finally, we characterized the effects of a single phage and the whole cocktail on the natural floral microbiome of pear blossoms through 16S community sequencing to evaluate potential safety implications of the phage application. Our results are in line with recent results from a separate, commercially available phage cocktail applied in apple trees (24) and emphasize that phages can be applied safely and effectively within the fire blight disease system.

## RESULTS

### The FireFighter phage cocktail significantly reduces symptom development as determined in an *in vitro* blossom assay

**Fig 1:**
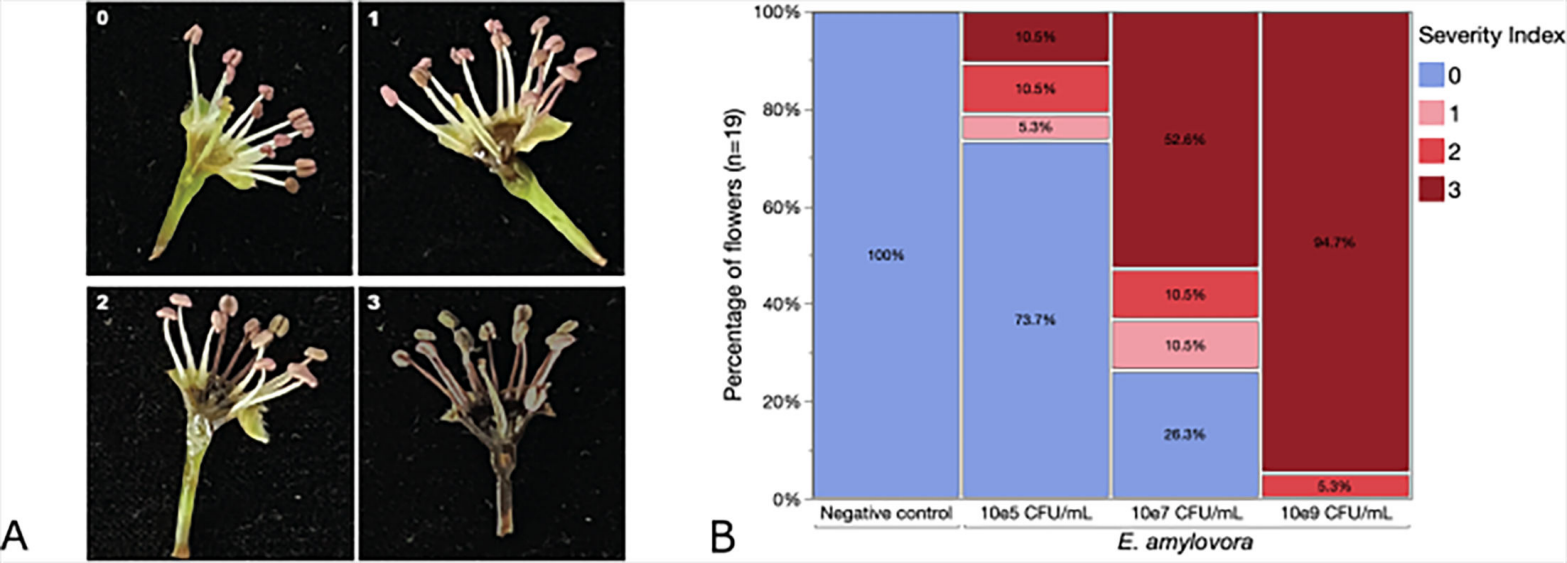
Optimization of an *in vitro* blossom assay to quantify disease progression in pear blossoms. (**A**) SI in pear blossoms 4 days post-inoculation of *E. amylovora*. SI 0: negative control; SI 1: mild discoloration of the receptacle and production of brown biofilm in the center of the flower; SI 2: dark discoloration of the receptacle, production of biofilm, and spread of the disease symptoms in other parts of the flower such as stamen filaments; and SI 3: stark discoloration of the receptacle, stamen filaments, biofilm production, and spread of the disease into the vascular tissues. (**B**) Mosaic plot summarizing the SI developed 4 days post-inoculation at 10^5^ CFU/mL, 10^7^ CFU/mL, and 10^9^ CFU/mL (*n* = 20 flowers per condition collected from two different trees).

Blossoms are a critical entry point for *E. amylovora* to initiate infection and cause fire blight symptoms during the spring season. To quantify the disease progression in pear blossoms, we adapted an *in vitro* blossom assay (28–30) using environmentally collected Callery pear blossoms to screen the optimal pathogen concentration and established a scoring system to score disease severity as a symptom index (SI; Fig. 1A): no visible discoloration of the receptacle (SI 0); mild discoloration of the receptacle and production of brown biofilm in the center of the flower (SI 1); dark discoloration of the receptacle with biofilm production and spread of the disease symptoms in other parts of the flower such as stamen filaments (SI 2); and stark discoloration of the receptacle, stamen filaments, biofilm production, and spread of the disease into the vascular tissues (SI 3). Only a minor fraction of the blossoms sprayed with a concentration of 10^5^ CFU/mL (10^5^ CFU/flower) developed disease symptoms 3 days after inoculation (5% SI 1, 10.5% SI 2, and 10.5% SI 3; Fig. 1B). Increasing the concentration to 10^7^ CFU/mL resulted in 74% of the flowers developing symptoms (10.5% SI 1, 10.5% SI 2, and 53% SI 3) and at a concentration of 10^9^ CFU/mL, all flowers showed severe disease symptoms (5% SI 2 and 95% SI 3).

**Fig 2:**
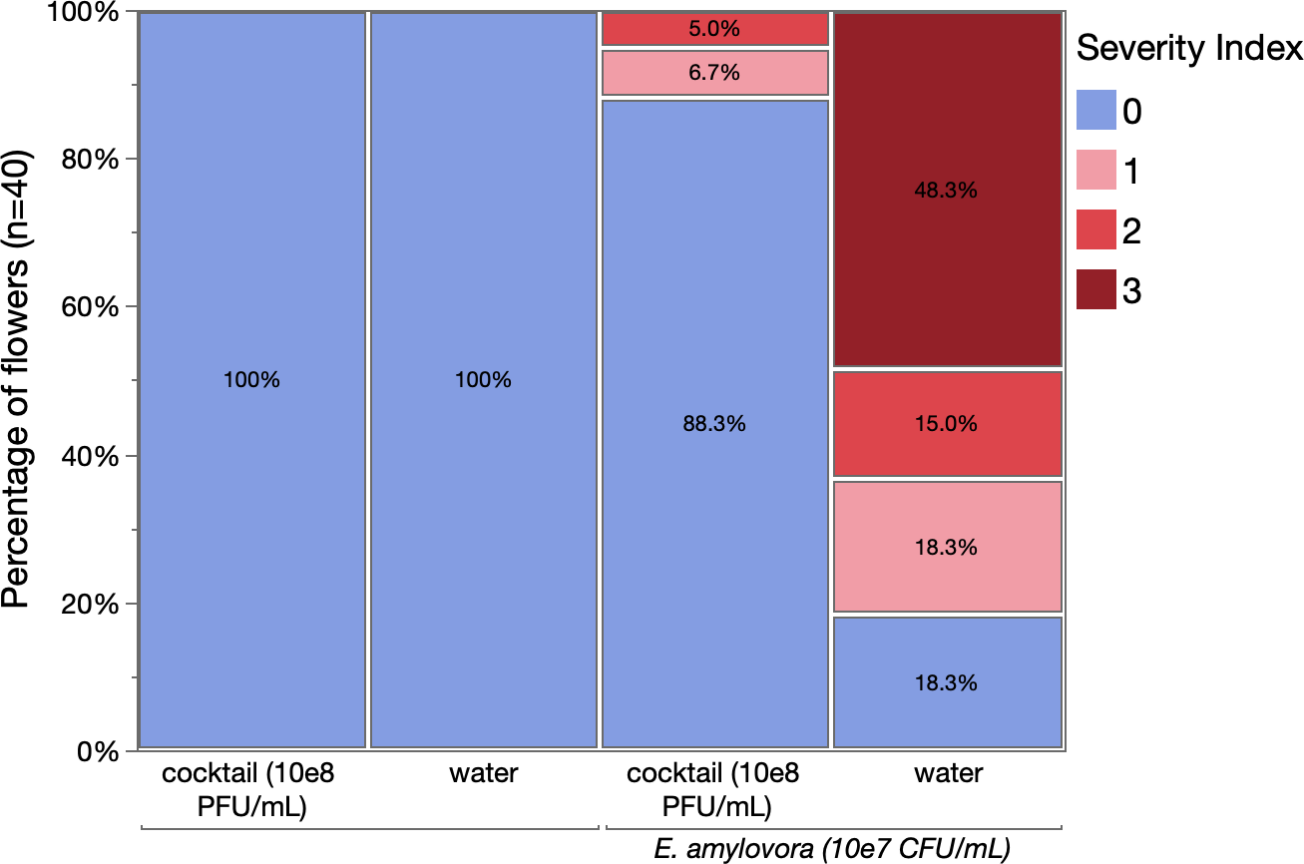
Mosaic plot summarizing the efficacy of the FireFighter phage cocktail in mitigating fire blight in pear blossoms: negative control (phosphate-buffered saline [PBS] and sterile water), phage cocktail and PBS, *E. amylovora* and sterile water, and *E. amylovora* and phage cocktail (*n* = 40 blossoms per tree per condition, six trees total; Wilcoxon test with correction for multiple comparison, *P*-value < 0.001).

Leveraging these results, we evaluated the potential of the FireFighter phage cocktail to reduce disease in pear blossoms. As such, flowers were sprayed with the phage cocktail at a final concentration of 10^8^ PFU/mL prior to an inoculation with *E. amylovora* at a concentration of 10^7^ CFU/mL. As illustrated in Fig. 2 (panels A and B), non-treated blossoms developed severe disease symptoms in sharp contrast to blossoms that were treated with the FireFighter phage cocktail (Wilcoxon test with corrections for multiple comparisons, *P*-value < 0.001). We could not find a significant effect of the location where the flowers were collected on the disease severity (Wilcoxon test, *P*-value = 0.5356). No significant differences were observed between the uninoculated blossoms and those treated with phage prior to bacterial inoculation, suggesting that the phage cocktail strongly reduced symptom development in the flowers.

### Non-symptomatic blossoms harbor increased diversity in community composition in the presence of *E. amylovora* after phage treatment

**Fig 3:**
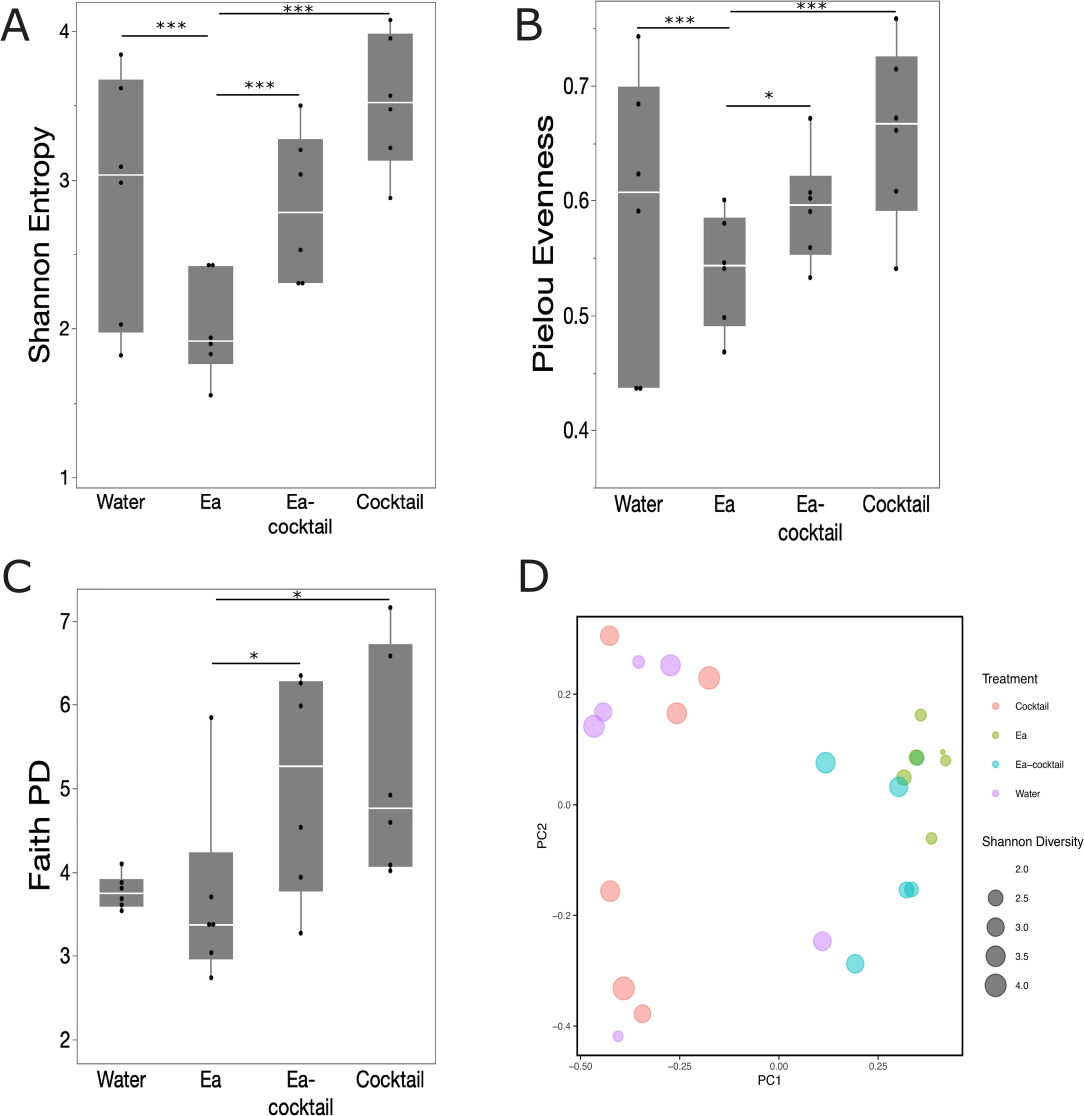
16S community sequencing of healthy blossoms three days after incubation. Quantile box plots of the alpha diversity ([**A**] Shannon, [**B**] Pielou Evenness, and [**C**] Faith PD) of the microbial community per treatment (*n* = 6 blossoms per condition, Kruskal-Wallis with corrections for multiple comparison, *P*-value < 0.05 [*], <0.02 [**], and <0.01 [***]). (**D**) Principal component analysis of the beta diversity (Bray-Curtis dissimilarity). The size of the data points is determined by the Shannon diversity of the respective community. The treatments are depicted in different colors: in green are microbiomes collected from blossoms treated with phage in the absence of *E. amylovora*, in teal microbiomes from blossoms inoculated with *E. amylovora* in the absence of phage, in blue microbiomes from blossoms treated with phage prior to an inoculation with *E. amylovora*, and in purple communities collected from the negative control.

Next, we assessed the diversity and community composition of the microbial communities after externally inoculating *E. amylovora* and compared them between the different treatments (Fig. 3). Here, we opted to assess non-symptomatic rather than symptomatic flowers as the communities of the latter were heavily dominated by *E. amylovora*. We observed a significant overall effect of phage/bacterial treatment on the richness of the microbial community based on both the Shannon index (Kruskal-Wallis test, *P*-value = 0.01) and Faith PD (*P*-value = 0.017). Comparing the different treatments more closely, we observed a significant reduction in the alpha diversity (Kruskal-Wallis test with correction for multiple comparison) between blossoms that were inoculated with *Ea* compared to blossoms that were treated with the phage cocktail in the absence of *Ea* and in buffer conditions (Pielou Evenness *P*-value 0.025, Faith PD *P*-value 0.025, and Shannon *P*-value 0.003; Fig. 3A through C). Based on the Shannon index and Faith PD metrics, treating blossoms with the FireFighter phage cocktail in the presence of *Ea* significantly increased the alpha diversity compared to flowers with Ea alone (Kruskal-Wallis test with correction for multiple comparison, *Ea* vs *Ea*-cocktail, *P*-value = 0.025 and *P*-value = 0.05, respectively). Together with a significant increase in the richness of the community after inoculating the cocktail in the absence of *Ea* (Kruskal-Wallis test with correction for multiple comparison, water vs cocktail, Faith PD *P*-value 0.01), these results suggest that the cocktail increased the overall diversity of the floral microbiome despite the presence of the pathogen.

**Fig 4:**
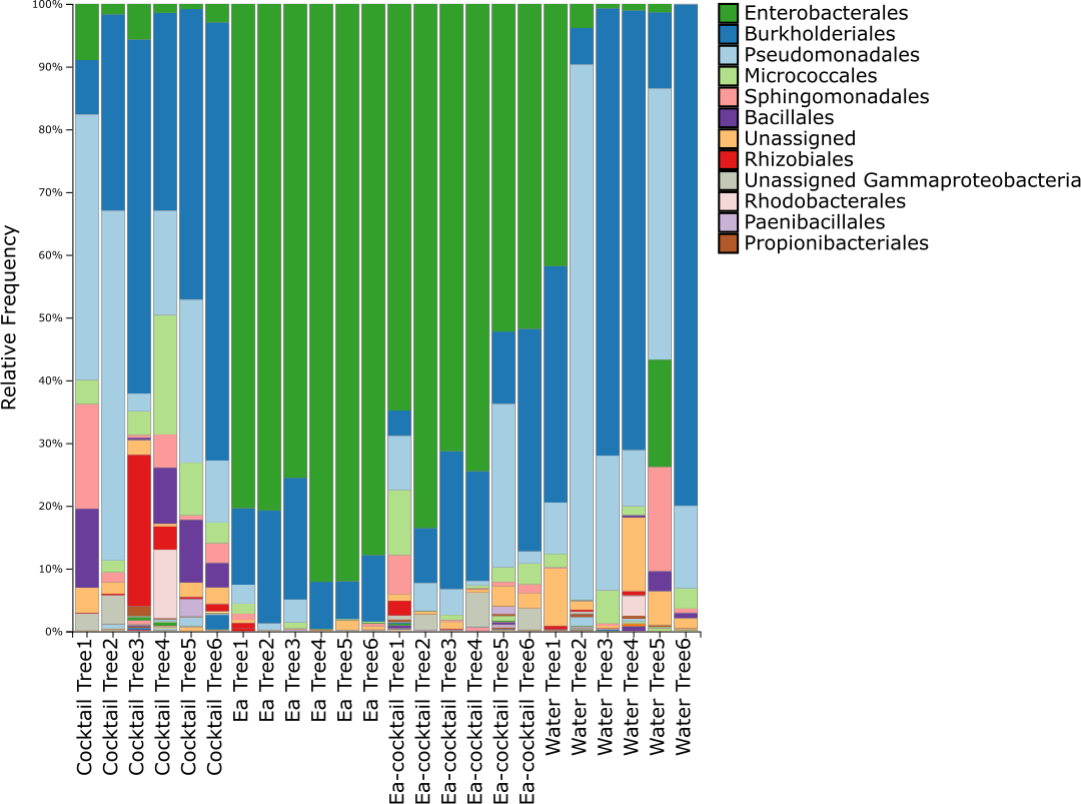
Taxonomic bar plot depicting the relative frequency of amplicon sequence variants found in healthy flowers externally inoculated with *E. amylovora* organized by treatment. From left to right: cocktail without the external inoculation of *E. amylovora*, *E. amylovora* without phage treatment, *E. amylovora* with phage cocktail, and sterile water.

Across the different treatments, we also found a significant shift in community composition as measured by beta diversity (Bray-Curtis dissimilarity) between the groups (PERMANOVA *P*-value = 0.001; Fig. 3D). More specifically, we distinguished three levels of significance based on the beta diversity: group A consisting of blossoms treated with water and the phage cocktail in absence of *Ea*, group B including flowers treated with the phage cocktail in presence of *Ea*, and group C grouping the flowers that were inoculated with *Ea* in the absence of phage (Pairwise PERMANOVA with corrections for multiple comparisons, *P*-value < 0.05; Fig. 3D) which is further illustrated in the relative abundance (Fig. 4). When *E. amylovora* is externally inoculated, it becomes the dominant member of the microbial community. Conversely, without the external inoculation of *E. amylovora*, Pseudomonadales and Burkholderiales are the most abundant members of the Callery pear blossom microbiome. Based on quantitative PCR (qPCR) targeting *E. amylovora*, we found a significant effect of externally inoculating *E. amylovora* (ANOVA, *P*-value < 0.001), but we could not identify a significant difference in the concentration of *E. amylovora* between the treated and non-treated blossoms (Fig. S1, Tukey test with corrections for multiple comparisons, *P*-value > 0.05).

### The FireFighter phage cocktail does not impact overall abundance, richness, or community composition of the floral microbiome

**Fig 5:**
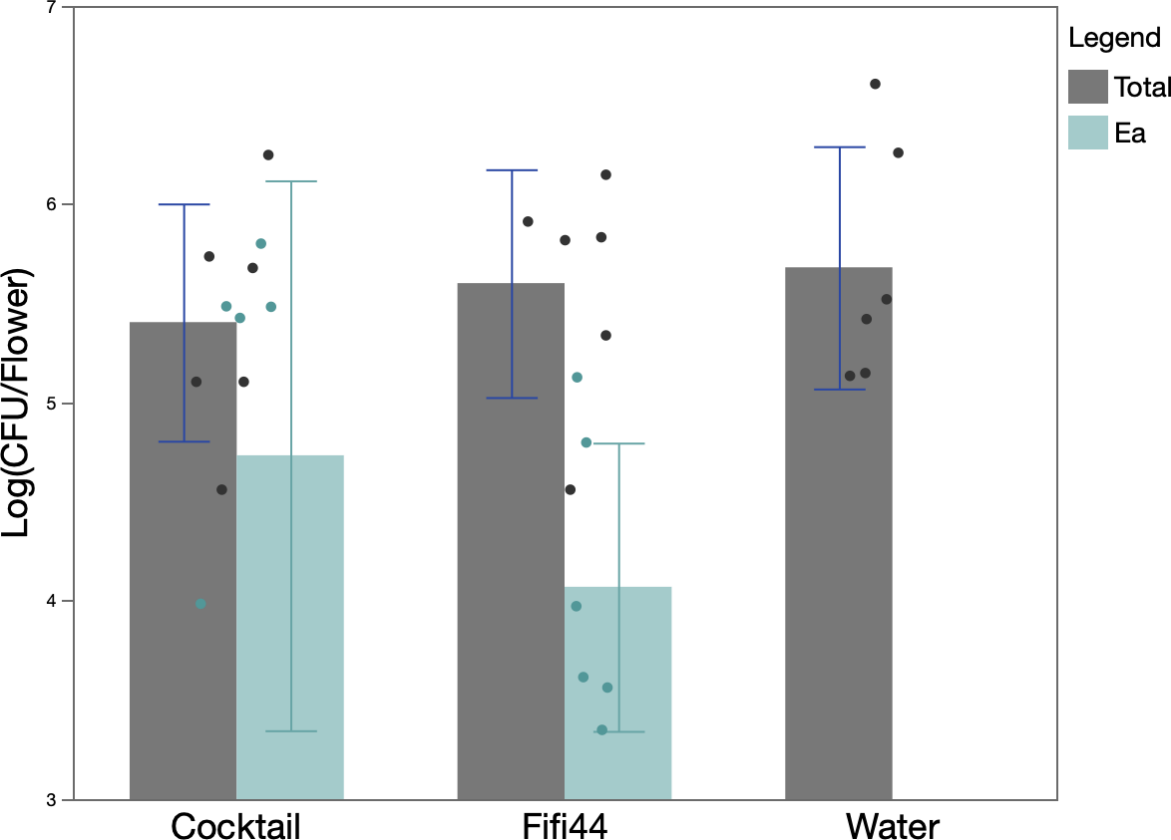
The mean of the total bacterial abundance (gray) of the culturable fraction of the microbial community and *E. amylovora* (teal) as determined with qPCR after a treatment with the full phage cocktail, phage Fifi44, and without phages (water) expressed as CFUs per flower (*n* = 6 flowers per tree [six trees total]). Error bars represent the SE on the data.

In order to assess the safety of the phage cocktail, we evaluated its impact on the natural floral microbiome. As such, we determined the number of CFUs per flower of the natural microbial community, as well as the concentration of *E. amylovora* in the flowers using qPCR (Fig. 5). Across the different trees from which the flowers were collected, we observed that the total bacterial concentration varied between 10^5^ and 10^7^ CFU/flower. Using a linear mixed-effects model followed by ANOVA, we could not distinguish a significant effect of treatment, tree, or the interaction term (tree × treatment) on the model. Based on the qPCR, there was a relatively high concentration of *E. amylovora* DNA in the flowers after phage treatment, which is likely explained by the presence of *E. amylovora* DNA in the phage lysate, as observed without the external inoculation of *E. amylovora*. Without the external inoculation of *E. amylovora* or phage suspension*,* there were no detectable copies of *E. amylovora* detectable with our qPCR (number of copies dropped below the limit of detection).

**Fig 6:**
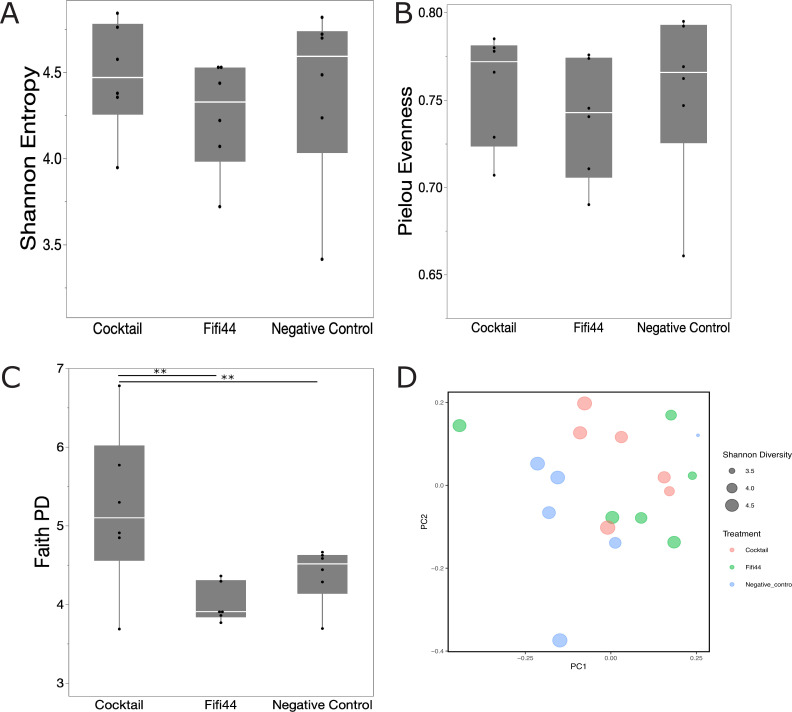
16S community composition of blossoms that were treated with a single phage (Fifi44), the full phage cocktail, and without phage treatment (negative control, sterile water). (**A–C**) Quantile box plots of the alpha diversity (Shannon Entropy, Pielou Evenness, and Faith PD, respectively) for the communities per treatment (six blossoms per condition, Kruskal-Wallis with correction for multiple comparison, *P*-value < 0.02 [**]). (**D**) Principal component analysis of the beta diversity (Bray-Curtis dissimilarity). The size of the data points is determined by the Shannon diversity of the respective community. The treatments are depicted in different colors: in red are microbiomes collected from blossoms treated with the full FireFighter phage cocktail*,* in green microbiomes from blossoms treated with Fifi44, and in blue communities collected from the negative control.

**Fig 7:**
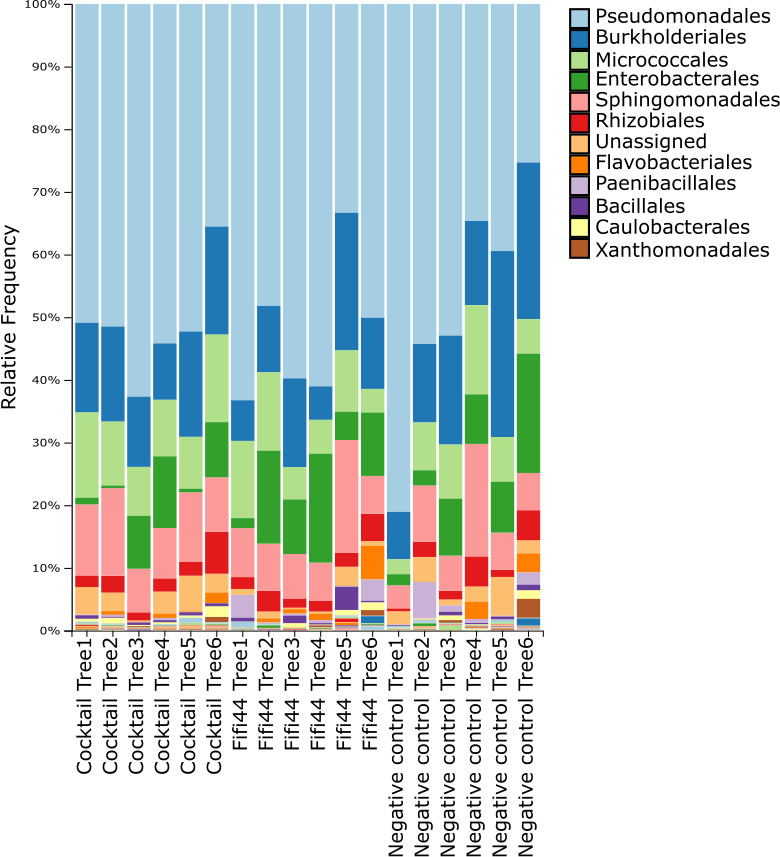
Taxonomic bar plot of the relative frequency of the amplicon sequence variants detected in the natural blossom microbiome with and without the application of the phage cocktail or a single-phage application with Fifi44. From left to right: application of the five-phage cocktail, single-phage application of Fifi44, negative control without the application of phage.

Next, we evaluated the influence of the phage applications on the overall richness and community composition of the floral microbiome. Across the different treatments, we found no significant impact of the FireFighter phage cocktail nor a single phage from this cocktail (Fifi44) on the alpha diversity of the community (Shannon and Pielou Evenness, Fig. 6A and B, respectively). Based on the Faith PD metric, we observed a significant increase in the richness after applying the FireFighter phage cocktail (Kruskal-Wallis test with correction for multiple comparison, *P*-value < 0.05). We did not observe any significant shifts in the beta diversity (PERMANOVA, *P*-value = 0.536; Fig. 6D). Looking more closely at the different taxonomic groups, the most dominant member of the Callery pear blossom microbiome is the *Pseudomonadales*, followed by the *Burkholderiales* and *Micrococcales* (Fig. 7).

## DISCUSSION

Fire blight caused by *E. amylovora* poses a global threat to apple and pear production. In recent years, the disease has spread to new regions in the world, causing issues to local farming communities and the general availability of fruit in specific regions. In particular, the recent outbreak of fire blight in South Korea has affected more than 744 commercial orchards as of 2020 (2). This has resulted in an urgent need for mechanisms of disease control, including the use of phages. Like any other bacterial disease, fire blight can be considered a dysbiosis of the microbial community in which the pathogen, *E. amylovora*, becomes the dominant member of this community. Recent insight into the apple floral microbiome associated with fire blight shows that in healthy flowers, the microbiome is primarily dominated by *Pantoea* spp. and *Lorielloppsis cavernicola*. In contrast, the dysbiotic community consists primarily of *Pseudomonas* spp*.*, *Salmonella* spp*.*, and *Erwinia* spp., with a high abundance of *E. amylovora* (31). Additionally, fire blight is characterized by a significantly decreased alpha diversity (Shannon diversity index and Pielou evenness) alongside a shift in the beta diversity (Bray-Curtis) (8, 31). In line with this, our *ex planta* system confirmed that the external inoculation of *E. amylovora* to naturally collected Callery pear blossoms significantly decreased the alpha diversity of the floral microbiome, and we calculated a significant shift of the community composition in non-symptomatic blossoms (Fig. 5), as similarly described by Cui and colleagues during field studies (8). Hence, we consider our *ex planta* bioassay a valuable tool to evaluate the impact of biocontrol agents such as bacteriophages on disease progression as well as the natural floral microbiome.

In our study, we looked at the efficacy of the FireFighter phage cocktail to control *E. amylovora* in pear blossoms, as well as its effects on the overall microbial community to evaluate the safety of the phage cocktail. We showed that under a relatively high pathogen pressure, the phage cocktail was able to significantly improve blossom health *in vitro* (Fig. 2). These results underscore the potential of this cocktail as an effective biocontrol tool to mitigate fire blight during blossoming, as recently summarized by Gayder and colleagues (22). Because the efficacy of *E. amylovora* phages is impacted by the production of extracellular polymeric substances, future research should look at the performance of the phage cocktail to control high amylovoran-producing *E. amylovora* isolates (32).

We further evaluated the effects of this cocktail on the larger microbial community in the presence and absence of the pathogen. As such, we demonstrated that the phage application partially restored both the richness and community composition of the floral microbiome despite the presence of the pathogen and hence alleviated the dysbiosis caused by *E. amylovora* (Fig. 5). Similar observations were made when microcosms were inoculated with *Xanthomonas campestris* pv. *campestris*, and the effects of two phages as candidates for biocontrol were evaluated on the soil microbiome. While the phages were found to reduce the host biomass in soil samples, they had a minimal impact on the richness and composition of the soil microbiome (33). Budding research on the effects of phages on the plant-associated microbial community demonstrated that after a phage application, the alpha diversity of the rhizosphere microbiota of tomato in greenhouse conditions did not significantly change (34). Similarly, no significant effects of phages on either the alpha or beta diversity of the soil microbiome were detected after applying *Xanthomonas campestris* pv. *campestris* phages in both controlled environments as well as field conditions (33). These results were further confirmed by Gdanetz and colleagues, as well as they described a reduction of the abundance of *Erwinia* after the primary application of AgriPhage without any further implications to the microbial community in apple (24).

An important open question in the application of phages is their overall safety and off-target effect on other members of the microbial community, and this might be particularly important in the case of multi-phage cocktails. To this end, we evaluated the effects of a single member of the FireFighter phage cocktail compared to a five-member, diverse phage cocktail on the natural floral microbiome of Callery pear in the absence of the host. Our results suggest that there is a minimal impact of the phages on the total abundance of the culturable fraction of the microbial community, as well as both the richness and composition of the full floral microbiome. Moreover, there was no difference between the single phage and phage cocktail on the non-target microbial community despite the wide diversity of the phages used. Interestingly, there was a significant increase in the alpha diversity metrics (Faith’s PD, which is calculated based on the branch lengths of the taxonomic tree generated using all of the amplicon sequence variants [ASVs]) (35). This supports the hypothesis that phage cocktails might increase the taxonomic richness of the microbial community, as has been previously observed for whole viral transplants in tomato plants (36). Similar results have also been described for a single phage, KMM4, on the *Aurelia aurita* microbiome, although other phages in the same study were found to have a negative effect on the richness, including a cocktail composed of four phages including KMM4 (37). While our study suggests a minimal impact of bacteriophages on the floral microbiome of Callery pear blossoms based on universal bacterial markers (16S), we are limited in our ability to make predictions on the effects of phages on microbiome function. In the future, studies employing metagenomics might shed better light on whether there are strain-level effects of phages and/or whether phage presence alters specific gene presence/absence.

### Conclusion

With the increasing need to find alternative disease control strategies in agriculture, the application of phages is rapidly gaining in popularity. Here, we extend recent work to show that both a single phage and a diverse, five-phage cocktail can each vastly reduce disease symptoms of fire blight in pear blossoms inoculated with *E. amylovora* without altering the resident microbiome of the flowers. The lack of off-target effects observed, as well as some evidence for increased microbiome diversity in the presence of the phage, suggests that phage application in this case is unlikely to reduce other bacterial competition effects in the plant that might also be preventing pathogen establishment. We also show that the *ex planta* blossom assay shows great promise as a semi-natural setting for the measurement of disease control by phages. In combination with a recent field trial on apples from a commercial phage cocktail in the US (24), our data add to the small but growing evidence that deliberate and well-defined phage applications are unlikely to have an impact beyond the target bacterial pathogens they are used to treat.

## MATERIALS AND METHODS

### Bacterial and bacteriophage manipulations

*E. amylovora* isolate T8 was isolated from Callery pear in Berkeley, California, and used for phage propagation and inoculation assays. The bacteria were cultured in King’s Broth (KB) with shaking at 150 rpm or cultured on KB agar plates at 28°C. Four bacteriophages, Fifi011, Fifi44, Fifi287, and Fifi318, were used in this study. Each bacteriophage was propagated individually with *E. amylovora* T8 strain for 24 h at 28°C in liquid KB medium by inoculating an exponentially growing culture (optical density of 0.3) with a multiplicity of infection of 0.1. Following centrifugation at 6,000 × *g* at 4°C for 20 min, the supernatant was filtered through a 0.22 µm polyvinylidene fluoride (PVDF) syringe filter (Millipore, Bedford, MA, USA) for the removal of bacterial cells and other debris. The phage lysate was concentrated using a 100 kD centrifugal filtration device (Millipore, Amicon Ultra Centrifugal Filters) and resuspended with sodium chloride–magnesium sulfate (SM) buffer (50 mM/L Tris-HCl, 100 mM NaCl, and 10 mM MgSO_4_; pH 7.5). The phage lysate (10^10^ PFU/mL as determined by plaque assays on Ea T8) was stored at 4°C for further use. Each phage was diluted to ±0.25 × 10^8^ PFU/mL in sterile water and combined to the final concentration of 10^8^ PFU/mL before spraying on pear blossoms.

### Efficacy of the phage cocktail to reduce symptom development in Callery pear blossoms and effect of phages on the microbial community after inoculation of *E. amylovora*

Young Callery pear blossoms 1 day after opening (blossoms just out of the popcorn stage with pink stamen) were harvested from six trees in February 2023 in distinct geographical locations of Berkeley, California. After collection, the flowers (*n* = 10/plate) were placed in deep-well water agar plates (mQ supplemented with 10 g/L agar). Depending on the condition, the flowers were first treated with a phage suspension consisting of either Fifi44 or the entire phage cocktail at 10^8^ PFU/mL diluted in sterile water using 10 mL per plate sprayed with a sterile polypropylene fingertip pump misters (The Cary Company, part number 30WP24, autoclaved in aluminum foil) or sterile water in case of the control (six plates per condition, flowers collected from 6 trees, 10 flowers per tree). The bacterial inoculum was prepared by resuspending *E. amylovora* T8 grown on KB agar plates in phosphate-buffered saline (PBS) and diluted to a final concentration of 10^5^, 10^7^, and 10^9^ CFU/mL in PBS and sprayed over the flowers (10 mL per plate using a sterile hand sprayer per 10 flowers). In the case of the negative control, PBS was sprayed over the flowers. The plates were sealed with parafilm to maintain high humidity and incubated for 3–4 days at 28°C. Non-symptomatic blossoms (*n* = 6 per condition across the six trees) were collected and homogenized in PBS using sterile pestles. DNA was extracted from the homogenized flowers (500 µL) using the Qiagen PowerSoil Pro kit according to the manufacturer’s guidelines.

### Evaluating the effect of the phage cocktail on the natural microbial community in Callery pear blossoms

The effects of the phage cocktail on the natural microbial community in the absence of externally inoculated *E. amylovora* were evaluated by spraying Callery pear blossoms (*n* = 10, just out of the popcorn stage) collected from six trees in Berkeley, CA, and weighed. The blossoms were placed in deep-well water agar plates (*n* = 10/plate) and sprayed with either Fifi44, the cocktail (Fifi011, Fifi44, Fifi287, and Fifi318), or sterile water using sterile polypropylene fingertip pump misters (10 mL per plate). Blossoms here pooled according to the tree from which they were collected, suspended in a phosphate-glycerol buffer (0.03M NaH_2_PO_4_, 0.07 M Na_2_HPO_4_·7H_2_O, 1:10 vol% glycerol), and homogenized using sterile pestles. A dilution series was plated on KB agar plates and incubated for 48 h to obtain the total concentration of the culturable fraction of the bacterial community. DNA was extracted from the pooled blossom homogenates (500 µL) using the Qiagen PowerSoil Pro kit as described by the manufacturer.

### DNA extraction and 16S community sequencing

DNA concentrations were quantified using the Qubit dsDNA HS Assay kit. The V5–V7 region of the 16S rRNA gene was sequenced according to Meyer and colleagues (38). In short, the V5–V7 region was amplified using the 799F (5′–AACMGGATTAGATACCCKG–3′) and 1193R (5′–ACGTCATCCCCACCTTCC–3′) primers to exclude as much chloroplast and mitochondrial contamination as possible (38). The amplicons were diluted 8:1 and amplified for an additional 9 cycles to add sample-specific barcodes, followed by a Qubit quantification, pooling, and sequencing on the MiSeq (paired-end 300) platform.

### qPCR detection of *E. amylovora*

The DNA concentration of *E. amylovora* from blossom homogenates was quantified with qPCR using primers hpEaF (5′-CCGTGGAGACCGATCTTTTA-3′) and hpEaR (5′-AAGTTTCTCCGCCCTACGAT-3′; Integrated DNA Technologies) in triplicate, targeting the AMY1267 gene (39). Reactions were run on an Applied Biosystems StepOne Real-Time PCR System with Thermo Scientific Maxima SYBR Green qPCR Master Mix. Each 25 µL reaction contained 12.5 µL SYBR Master Mix, 0.3 µM of each primer, 10 nM ROX reference dye, 2.5 µL DNA template, and 8.95 µL UltraPure Nuclease-Free Water. Thermal cycling consisted of an initial denaturation at 95°C for 10 min, followed by 40 cycles of 95°C for 15 s and 60°C for 60 s. Standards were run in triplicate on each plate using 8 points of 1:10 serial dilutions of *E. amylovora* T8 cultures grown overnight in KB. Dilutions were plated on KB agar to determine CFU per milliliter, and the average cycle threshold (Ct) values from triplicate reactions were used to generate standard curves (*R*² ≥ 0.998), which enabled us to calculate the CFU per milliliter and normalize according to the number of flowers homogenized in 1 mL of sterile buffer. Each flower homogenate sample was run in triplicate, and Ct values were used to calculate CFU per milliliter using the standard curve.

### Bioinformatic and statistical analysis

The 16S rRNA amplicons were analyzed by means of the QIIME2 pipeline (40). The reads were processed using Deblur (41) and trimmed to a length of 150 bp and quality filtered. Chimeric reads were removed from the data set. The taxonomy of the sequences was determined by means of the classify-skylearn function using the SILVA (v138) SSURef NR99 full-length sequences database (42–44). Remaining chloroplast and mitochondrial ASVs were filtered using the taxa filter-table function in QIIME2, excluding both mitochondria and chloroplasts (--p-exclude mitochondria, chloroplast). Community matrices were rarefied according to Meyer and colleagues (38). In short, the matrices were rarefied to 8,000 counts per sample and averaged to account for sampling differences. The phylogenetic diversity analysis was performed using the align-to-tree-mafft-fasttree function with 1,000 bootstraps. The diversity metrics of the samples were calculated using the “diversity core-metrics-phylogenetic” command in QIIME2 (45). These included Shannon’s diversity index, Faith’s Phylogenetic Diversity, and Evenness, as well as the Bray-Curtis distance for the alpha and beta diversity, respectively. Differences among the community richness of the different conditions were assessed using a Kruskal-Wallis test and Kruskal-Wallis test with correction for multiple comparisons. Differences in beta diversity were assessed using a PERMANOVA test. The alpha diversity metrics were visualized using JMP Pro (v16), and the principal component analysis was visualized using the qiime2R (v0.99) package in R (46).

## Data Availability

The sequencing data from this project are available in NCBI with accession number PRJNA1249099.

## SUPPLEMENTAL MATERIAL

Figure S1 aem.01598-25-s0001.docx Concentration of *E. amylovora* in non-symptomatic flowers after external inoculation, as determined by qPCR performed in triplicate on the DNA isolated from the flower homogenates.

ASM does not own the copyrights to Supplemental Material that may be linked to, or accessed through, an article. The authors have granted ASM a non-exclusive, world-wide license to publish the Supplemental Material files. Please contact the corresponding author directly for reuse.

## References

[1] Pedroncelli A, Puopolo G. 2024. This tree is on fire: a review on the ecology of Erwinia amylovora, the causal agent of fire blight disease. J Plant Pathol 106:823–837. doi:10.1007/s42161-023-01397-y

[2] Jik Lee H, Woo Lee S, Suh S, Hyun I. 2022. Recent spread and potential pathways for fire blight in South Korea. EPPO Bulletin 52:135–140. doi:10.1111/epp.12835

[3] Slack SM, Zeng Q, Outwater CA, Sundin GW. 2017. Microbiological examination of Erwinia amylovora Exopolysaccharide ooze. Phytopathology 107:403–411. doi:10.1094/PHYTO-09-16-0352-R 28045342

[4] Roussin-Léveillée C, Rossi CAM, Castroverde CDM, Moffett P. 2024. The plant disease triangle facing climate change: a molecular perspective. Trends Plant Sci 29:895–914. doi:10.1016/j.tplants.2024.03.004 38580544

[5] McGuire D, Pinto F, Costa T, Cruz J, Sousa R, de Sousa ML, Martins C, Gama-Carvalho M, Tenreiro A, Tenreiro R, Cruz L. 2024. Fire4CAST – a new integrative epidemiological forecasting model for the accurate prediction of infection risk and effective control of fire blight in Pyrus orchards. J Plant Pathol 106:953–966. doi:10.1007/s42161-024-01622-2

[6] Cui Z, Huntley RB, Schultes NP, Kakar KU, Yang CH, Zeng Q. 2021. Expression of the type III secretion system genes in epiphytic Erwinia amylovora cells on apple stigmas benefits endophytic infection at the hypanthium. Mol Plant Microbe Interact 34:1119–1127. doi:10.1094/MPMI-06-21-0152-R 34698527

[7] Steven B, Huntley RB, Zeng Q. 2018. The influence of flower anatomy and apple cultivar on the apple flower phytobiome. Phytobiomes Journal 2:171–179. doi:10.1094/PBIOMES-03-18-0015-R

[8] Cui Z, Huntley RB, Zeng Q, Steven B. 2021. Temporal and spatial dynamics in the apple flower microbiome in the presence of the phytopathogen Erwinia amylovora. ISME J 15:318–329. doi:10.1038/s41396-020-00784-y 33024293 PMC7853089

[9] Koskella B, Hernandez CA, Wheatley RM. 2022. Understanding the impacts of bacteriophage viruses: from laboratory evolution to natural ecosystems. Annu Rev Virol 9:57–78. doi:10.1146/annurev-virology-091919-075914 35584889

[10] Zlatohurska M, Gorb T, Romaniuk L, Shenderovska N, Faidiuk Y, Zhuminska G, Hubar Y, Hubar O, Kropinski AM, Kushkina A, Tovkach F. 2023. Broad-host-range lytic Erwinia phage key with exopolysaccharide degrading activity. Virus Res 329:199088. doi:10.1016/j.virusres.2023.199088 36907559 PMC10194208

[11] Thompson DW, Casjens SR, Sharma R, Grose JH. 2019. Genomic comparison of 60 completely sequenced bacteriophages that infect Erwinia and/or Pantoea bacteria. Virology (Auckl) 535:59–73. doi:10.1016/j.virol.2019.06.005 31276862

[12] Akremi I, Holtappels D, Brabra W, Jlidi M, Hadj Ibrahim A, Ben Ali M, Fortuna K, Ahmed M, Meerbeek BV, Rhouma A, Lavigne R, Ben Ali M, Wagemans J. 2020. First report of filamentous phages isolated from Tunisian orchards to control Erwinia amylovora. Microorganisms 8:1–17. doi:10.3390/microorganisms8111762 PMC7697814 33182526

[13] Born Y, Bosshard L, Duffy B, Loessner MJ, Fieseler L. 2015. Protection of Erwinia amylovora bacteriophage Y2 from UV-induced damage by natural compounds. Bacteriophage 5:1–5. doi:10.1080/21597081.2015.1074330 PMC4743488 26904378

[14] Parcey M, Gayder S, Castle AJ, Svircev AM. 2020. Molecular profile of phage infection: a novel approach for the characterization of Erwinia phages through qPCR. Int J Mol Sci 21:1–15. doi:10.3390/ijms21020553 PMC7014438 31952282

[15] Gayder S, Parcey M, Nesbitt D, Castle AJ, Svircev AM. 2020. Population dynamics between Erwinia amylovora, Pantoea agglomerans and bacteriophages: exploiting synergy and competition to improve phage cocktail efficacy. Microorganisms 8:1–18. doi:10.3390/microorganisms8091449 PMC7563384 32971807

[16] Kolozsváriné Nagy J, Schwarczinger I, Künstler A, Pogány M, Király L. 2015. Penetration and translocation of Erwinia amylovora-specific bacteriophages in apple - a possibility of enhanced control of fire blight. Eur J Plant Pathol 142:815–827. doi:10.1007/s10658-015-0654-3

[17] Boulé J, Sholberg PL, Lehman SM, O’gorman DT, Svircev AM. 2011. Isolation and characterization of eight bacteriophages infecting Erwinia amylovora and their potential as biological control agents in British Columbia, Canada. Canadian Journal of Plant Pathology 33:308–317. doi:10.1080/07060661.2011.588250

[18] Born Y, Fieseler L, Thöny V, Leimer N, Duffy B, Loessner MJ. 2017. Engineering of bacteriophages Y2::dpoL1-C and Y2::luxAB for efficient control and rapid detection of the fire blight pathogen, Erwinia amylovora. Appl Environ Microbiol 83:e00341-17. doi:10.1128/AEM.00341-17 28389547 PMC5452800

[19] Born Y, Fieseler L, Marazzi J, Lurz R, Duffy B, Loessner MJ. 2011. Novel virulent and broad-host-range Erwinia amylovora bacteriophages reveal a high degree of mosaicism and a relationship to Enterobacteriaceae phages. Appl Environ Microbiol 77:5945–5954. doi:10.1128/AEM.03022-10 21764969 PMC3165370

[20] Schwarczinger I, Kolozsváriné Nagy J, Künstler A, Szabó L, Geider K, Király L, Pogány M, Nagy JK, Kunstler A, Szabo L, Kiraly L, Pogany M. 2017. Characterization of Myoviridae and Podoviridae family bacteriophages of Erwinia amylovora from hungary - potential of application in biological control of fire blight. Eur J Plant Pathol 149:639–652. doi:10.1007/s10658-017-1214-9

[21] Born Y, Fieseler L, Klumpp J, Eugster MR, Zurfluh K, Duffy B, Loessner MJ. 2014. The tail-associated depolymerase of Erwinia amylovora phage L1 mediates host cell adsorption and enzymatic capsule removal, which can enhance infection by other phage. Environ Microbiol 16:2168–2180. doi:10.1111/1462-2920.12212 23944160

[22] Gayder S, Kammerecker S, Fieseler L. 2023. Biological control of the fire blight pathogen Erwinia amylovora using bacteriophages. J Plant Pathol 106:853–869. doi:10.1007/s42161-023-01478-y

[23] Holtappels D, Alfenas-Zerbini P, Koskella B. 2023. Drivers and consequences of bacteriophage host range. FEMS Microbiol Rev 47:fuad038. doi:10.1093/femsre/fuad038 37422441

[24] Gdanetz K, Dobbins MR, Villani SM, Outwater CA, Slack SM, Nesbitt D, Svircev AM, Lauwers EM, Zeng Q, Cox KD, Sundin GW. 2024. Multisite field evaluation of bacteriophages for fire blight management: incorporation of ultraviolet radiation protectants and impact on the apple flower microbiome. Phytopathology 114:1028–1038. doi:10.1094/PHYTO-04-23-0145-KC 37581441

[25] Roh E, Duffy ME, Ewool LM, Grose JH. 2025. Whole genome sequences of eight Erwinia amylovora phages isolated from South Korea. Microbiol Resour Announc 14:e0106224. doi:10.1128/mra.01062-24 39999472 PMC11984208

[26] Park J, Kim B, Song S, Lee YW, Roh E. 2022. Isolation of nine bacteriophages shown effective against Erwinia amylovora in Korea. Plant Pathol J 38:248–253. doi:10.5423/PPJ.NT.11.2021.0172 35678058 PMC9343912

[27] Kim B, Lee SY, Park J, Song S, Kim KP, Roh E. 2024. Bacteriophage cocktail comprising Fifi044 and Fifi318 for biocontrol of Erwinia amylovora. Plant Pathol J 40:160–170. doi:10.5423/PPJ.OA.01.2024.0005 38606446 PMC11016559

[28] Cecala JM, Landucci L, Vannette RL. 2025. Seasonal assembly of nectar microbial communities across angiosperm plant species: assessing contributions of climate and plant traits. Ecol Lett 28:e70045. doi:10.1111/ele.70045 39737670 PMC11687353

[29] Pusey PL. 1997. Crab apple blossoms as a model for research on biological control of fire blight. Phytopathology 87:1096–1102. doi:10.1094/PHYTO.1997.87.11.1096 18945005

[30] Mukhtar S, Hassani MA, Zarrillo T, Cui Z, Sundin GW, Zeng Q. 2024. The role of foraging pollinators in assembling the flower microbiota and transmitting the fire blight pathogen Erwinia amylovora . Environ Microbiol 26:e16702. doi:10.1111/1462-2920.16702 39389580

[31] Kong HG, Ham H, Lee MH, Park DS, Lee YH. 2021. Microbial community dysbiosis and functional gene content changes in apple flowers due to fire blight. Plant Pathol J 37:404–412. doi:10.5423/PPJ.NT.05.2021.0072 34365752 PMC8357563

[32] Roach DR, Sjaarda DR, Castle AJ, Svircev AM. 2013. Host exopolysaccharide quantity and composition impact Erwinia amylovora bacteriophage pathogenesis. Appl Environ Microbiol 79:3249–3256. doi:10.1128/AEM.00067-13 23503310 PMC3685245

[33] Fortuna KJ, Szoboszlay M, Holtappels D, Lavigne R, Tebbe CC, Wagemans J. 2023. Assessing the environmental biosafety of phage-based biocontrol applications. Biol Control 187:1–11. doi:10.1016/j.biocontrol.2023.105375

[34] Yang K, Wang X, Hou R, Lu C, Fan Z, Li J, Wang S, Xu Y, Shen Q, Friman VP, Wei Z. 2023. Rhizosphere phage communities drive soil suppressiveness to bacterial wilt disease. Microbiome 11:1–18. doi:10.1186/s40168-023-01463-8 36721270 PMC9890766

[35] Faith DP. 1992. Conservation evaluation and phylogenetic diversity. Biol Conserv 61:1–10. doi:10.1016/0006-3207(92)91201-3

[36] Morella NM, Gomez AL, Wang G, Leung MS, Koskella B. 2018. The impact of bacteriophages on phyllosphere bacterial abundance and composition. Mol Ecol 27:2025–2038. doi:10.1111/mec.14542 29457297

[37] Stante M, Weiland-Bräuer N, von Hoyningen-Huene AJE, Schmitz RA. 2024. Marine bacteriophages disturb the associated microbiota of Aurelia aurita with a recoverable effect on host morphology. Front Microbiol 15:1356337. doi:10.3389/fmicb.2024.1356337 38533338 PMC10964490

[38] Meyer KM, Muscettola IE, Vasconcelos ALS, Sherman JK, Metcalf CJE, Lindow SE, Koskella B. 2023. Conspecific versus heterospecific transmission shapes host specialization of the phyllosphere microbiome. Cell Host Microbe 31:2067–2079. doi:10.1016/j.chom.2023.11.002 38029741

[39] Gottsberger RA. 2010. Development and evaluation of a real-time PCR assay targeting chromosomal DNA of Erwinia amylovora. Lett Appl Microbiol 51:285–292. doi:10.1111/j.1472-765X.2010.02892.x 20666990

[40] Bolyen E, Rideout JR, Dillon MR, Bokulich NA, Abnet CC, Al-Ghalith GA, Alexander H, Alm EJ, Arumugam M, Asnicar F, et al.. 2019. Reproducible, interactive, scalable and extensible microbiome data science using QIIME 2. Nat Biotechnol 37:852–857. doi:10.1038/s41587-019-0209-9 31341288 PMC7015180

[41] Amir A, McDonald D, Navas-Molina JA, Kopylova E, Morton JT, Zech Xu Z, Kightley EP, Thompson LR, Hyde ER, Gonzalez A, Knight R. 2017. Deblur rapidly resolves single-nucleotide community sequence patterns. mSystems 2:e00191–16. doi:10.1128/mSystems.00191-16 28289731 PMC5340863

[42] Glöckner FO, Yilmaz P, Quast C, Gerken J, Beccati A, Ciuprina A, Bruns G, Yarza P, Peplies J, Westram R, Ludwig W. 2017. 25 years of serving the community with ribosomal RNA gene reference databases and tools. J Biotechnol 261:169–176. doi:10.1016/j.jbiotec.2017.06.1198 28648396

[43] Yilmaz P, Parfrey LW, Yarza P, Gerken J, Pruesse E, Quast C, Schweer T, Peplies J, Ludwig W, Glöckner FO. 2014. The SILVA and “All-species Living Tree Project (LTP)” taxonomic frameworks. Nucl Acids Res 42:D643–D648. doi:10.1093/nar/gkt1209 24293649 PMC3965112

[44] Quast C, Pruesse E, Yilmaz P, Gerken J, Schweer T, Yarza P, Peplies J, Glöckner FO. 2013. The SILVA ribosomal RNA gene database project: improved data processing and web-based tools. Nucleic Acids Res 41:D590–6. doi:10.1093/nar/gks1219 23193283 PMC3531112

[45] Robeson MS 2nd, O’Rourke DR, Kaehler BD, Ziemski M, Dillon MR, Foster JT, Bokulich NA. 2021. RESCRIPt: Reproducible sequence taxonomy reference database management. PLoS Comput Biol 17:e1009581. doi:10.1371/journal.pcbi.1009581 34748542 PMC8601625

[46] Jordan EB. 2018. qiime2R: Importing QIIME2 artifacts and associated data into R sessions. Available from: https://github.com/jbisanz/qiime2R

