# Supplementary material for: Impact of phage treatment on fire blight disease outcome and floral microbiome composition
Source: Appl Environ Microbiol. 2025 Oct 15;91(11):e01598-25. doi: 10.1128/aem.01598-25 (PMC12628803; doi:10.1128/aem.01598-25)
Supplement: Figure S1 — Concentration of E. amylovora in non-symptomatic flowers after external inoculation, as determined by qPCR performed in triplicate on the DNA isolated from the flower homogenates. [file aem.01598-25-s0001.docx]

**Supplementary figure**

Supplementary Figure 1: Concentration of E. amylovora in non-symptomatic flowers after external inoculation of Ea as determined by a qPCR performed in triplicate on the DNA isolated from the flower homogenates. Four different treatments were assessed (cocktail without Ea, Ea without cocktail, Ea with cocktail, and no external inoculation of Ea or phage cocktail). Based on an ANOVA analysis, there was a significant effect of externally inoculating E. amylovora to the flowers. However, there was no significant difference between the treated and the non-treated blossoms (Tukey test with corrections for multiple comparison, p-value >0.05).
